# Body size perceptions and preferences favor overweight in adult Saharawi refugees

**DOI:** 10.1186/s12937-018-0330-5

**Published:** 2018-02-09

**Authors:** Desire Alice Naigaga, David Jahanlu, Hanne Marit Claudius, Anne Karine Gjerlaug, Ingrid Barikmo, Sigrun Henjum

**Affiliations:** Oslo and Akershus University College of Applied Sciences, P.O. Box 4, St. Olavs plass, 0130 OSLO, Norway

**Keywords:** Overweight, Obesity, Saharawi refugees, Body size perception, Desired body size, Body discrepancy

## Abstract

**Background:**

Culture affects body image and body size perceptions from an early age and in many African countries, overweight has been associated with richness, health, strength, and fertility. The present study investigated body size perceptions and preferences in an African refugee population.

**Methods:**

The cross-sectional study was comprised of 180 and 175 randomly selected Saharawi women and men, respectively, between 18 and 80 years. Stunkard’s body figure scale was used to identify self-perceived body size, desired body size and desired body size in the opposite gender.

**Results:**

Approximately half of the participants had a correct self-perceived body size; among them 70% did not have a desire to have a smaller body size. Among women who preferred a body size corresponding to overweight in men, 77% also had a desired body size corresponding to overweight; compared to 43% for men. The youngest participants (18–25 years) were the least likely to overestimate their body size in comparison to the older participants (26–45 years and 46–80 years).

**Conclusion:**

We found an overall preference for an overweight body size, and a significant difference in body size perception associated with age.

## Background

Body image relates to “a person’s perceptions, feelings and thoughts about his or her body, and is usually conceptualized as incorporating body shape and size” [1]. As such, body image and body size perceptions are closely linked [2]. Culture affects body image and body size perceptions from an early age [3], and different ideals for body shape and weight are seen in different cultures [4, 5]. In many African and Arabic countries, overweight has been associated with richness, health, strength, and fertility [6, 7], whereas in the Western world, a slim body size is widely idealized [8]. However, shifting attitudes towards a more Western body ideal have been reported in non-Western countries [3]. This change could be attributed to globalization, urbanization, and a shift in dietary consumption and energy expenditure, defined as the nutrition transition [6, 9]. The study population in this paper is refugees from Western Sahara settled in the Algerian desert, aged 18–80 years. The participants were primarily recruited as part of a larger study on risk factors for non-communicable diseases among adult Sahrawian refugees. The informed consent included a statement allowing us to use the data on body size perceptions and preferences. Within this study population, socio-cultural traditions that favor large body size ideals are prevalent [10]. However, an acculturation towards the Western culture among younger refugees has been reported in previous studies of a similar population [11, 12]. It is therefore hypothesized that this acculturation to the Western ideal might influence the definition of ideal body size, especially among younger Saharawi refugees. This coexistence of “new western” and “traditional” cultures presents an interesting opportunity to investigate how body ideals are defined in this refugee population. The main objective of this paper is to describe body size perceptions and preferences in different age groups among adult Saharawi refugees.

## Methods

### Design, recruitment, and participants

A cross-sectional survey was carried out during September and October 2014 in five refugee camps near Tindouf, Algeria. The total population in all five camps was estimated to be approximately 165,000 [13]. In the present study, the eligible population were adults, both males and females, 18–80 years, living in one of the five refugee camps: Smara, El Aiune, Ausserd, Dakla, or Boujdor. The sample size was chosen based on an estimated prevalence of overweight and obesity of 50% and an absolute precision of 5% for the 80% confidence interval. Assuming an incomplete sampling from approximately 10% of the participants, we calculated a final desired sample size of 180 men and 180 women, as determined with Open Source Epidemiologic Statistics for Public Health [14]. Due to the unequal number of inhabitants in the five camps, a proportional-to-size method was used to select participants from each camp. A two-staged cluster sampling was performed based on gender and camp. The households were randomly selected by tossing a pen to decide the direction in which the research team was to drive upon leaving the dispensaries. The team drove toward the border of the camp, and each seventh household was selected. One man and one woman from each household were randomly selected. In households where men were not present, the woman was included and a man in the neighboring household was asked to participate. The final sample size consisted of 355 participants, 175 men and 180 women.

### Questionnaire

**Fig. 1:**
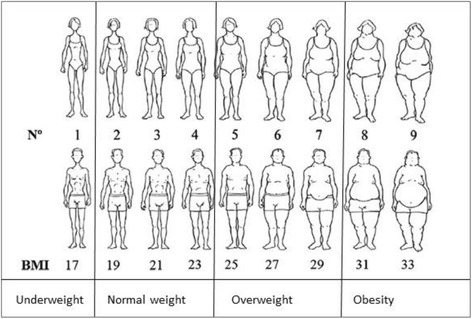
Body figure-scale [15]. BMI categorization by Acevedo et al 2014. BMI classes based on WHO recommendations [17]

A body figure scale developed by Stunkard (Fig. 1) was used to study body size perceptions and preferences [15]. Body mass index (BMI) was linked to each of the figures in the scale [16], and made it possible to categorize the figures into BMI subgroups according to the World Health Organization [17]. Data on “self-perceived body size” and “desired body size” were collected by showing the figure-scale to participants. For “preferred body size in opposite gender,” the figure-scale for men was shown to women and vice versa.

### Terms and clarifications

**Fig. 2:**
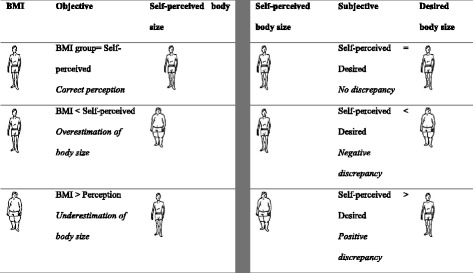
Explanation of terms used in the study

“Self-perceived body size” was defined as the body figure that participants themselves believed to have [18]. Participants expressed this by pointing to the body figure-scale. “Body size perception” was defined as a comparison between self-perceived body size and measured BMI. We used the term “underestimation” if one pointed at a smaller body size than his/her BMI subgroup, and “overestimation” if one pointed to a larger body size than the calculated BMI (Fig. 2). “Desired body size” was defined as the body size a person wished to have [19]. In some literature, it is also referred to as “ideal body size” [20] or “preferred body size.” If one indicated a size smaller than his/hers, it was defined as “positive body discrepancy;” if he/she indicated a larger size, then it was defined as “negative body discrepancy” [21–24]. These terms are further explained in Fig. 2.

We compared self-perceived body size with BMI. Studies have shown that BMI can be associated with body silhouettes [18]. Self-perceived body size was divided into four subgroups: underweight, normal weight, overweight, and obese, corresponding with BMI categorizations (Fig. 1).

### Anthropometric measures

To measure body weight, an electronic scale (Coline) and a solar–operated scale (SECA 890; Seca, Hamburg, Germany) were used. Participants were asked to remove shoes and an amount of clothes they were comfortable with. Half a kilo to 1 kilo was subtracted from body weight, based on measurements of traditional clothes. Height was measured using an electronic height rod (Soehlne professional ultrasonic height rod, MedicalExpo France). BMI categories set by the WHO were used [17], with one category set for obesity (BMI ≥ 30).

### Ethics

The study was approved by the Saharawi Health Authorities and The Norwegian Regional Committees for Medical and Health Research Ethics (2014/1155). Informed written consent was obtained from all participants and the study was conducted according to the guidelines provided in the Declaration of Helsinki.

### Statistical analyses

Statistical analyses were performed in IBM SPSS statistics version 22. Data is presented for the entire sample population and is presented based on gender and three age groups; 18–25 years, 26–45 years, and 46 years and older. For categorical data, we used 4 × 4 cross-tabulation and chi square statistics with *p* < 0.05 as significant level. We compared the following variables by cross tabulations: BMI and self-perceived body size, BMI and desired body size, self-perceived body size and desired body size, self-perceived body size and desired body size in opposite gender, desired body size and preferred body size in opposite gender.

## Results

The distribution of BMI categories by gender and age groups is presented in Table 1. Approximately 48% were classified as having normal weight, while 42% were overweight and obese. Significantly more women (60%) were overweight and obese compared to men (25%). In total, underweight was reported in only 10% of the participants. Among abnormal BMI categories, in the age group of 18–25 years, 16% were underweight, 16% were overweight, and 5% obese; corresponding results for the age group of 26–45 years, were 13%, 30%, and 14%, and for the age group 46 +, 3, 32, and 22%, respectively. The distribution of BMI among different age groups were significantly different (*p* = 0.003).

**Table 1:** Distribution of BMI categories by gender and age groups

		BMI categories n (% in rows)					
		Underweight	Normal weight	Overweight	Obese	Total	*p*-value**
Gender	Women	3 (1.9)	60 (37.2)	57 (35.4)	41 (25.5)	161 (100)	0.03
	Men	30 (17.8)	97 (57.4)	34 (20.1)	8 (4.7)	169 (100)	
	Total	33 (10.0)	157 (47.6)	91 (27.6)	49 (14.8)	330 (100)^a^	
Age groups	18–25	12 (16.2)	46 (62.2)	12 (16.2)	4 (5.4)	74 (100)	< 0.01
	26–45	17 (12.8)	58 (43.6)	40 (30.1)	18 (13.5)	133 (100)	
	46+	4 (3.3)	53 (43.0)	39 (31.7)	27 (22.0)	123 (100)	
	Total	33 (10.0)	157 (47.6)	91 (27.6)	49 (14.8)	330 (100)^a^	

^a^Missing values (*n* = 25), **p-values tested with chi-square

Distribution of participants’ body size perception by BMI categories and age groups is presented in Table 2. Approximately 49% (*n* = 154) had a correct body size perception. Of these, 45% (*n* = 69 of 154) were overweight/obese, and 48% of those who were classified as overweight/ obese (*n* = 65 of 135), underestimated their body size. According to gender, body size perception varied as follows: 77% of the participants that overestimated their body size were men. Among the women, 71% of those classified as being overweight had a correct perception of their body size, while only 10% of those classified as being obese had a correct perception of their body size. When categorized into the three age groups, analysis of the participants’ body size perception indicated that there was a significant difference (*p* = 0.003) among age groups and different classifications of body size perception. The majority of the participants with a correct body size perception were aged 26–45 years (47%); they also accounted for most participants who overestimated their body size (36%). The oldest participants (46–80 years) were found to underestimate their body size more than participants in the other age groups.

**Table 2:** Distribution of the self-perceived body size by BMI categories and age groups

		Body size perception n (% in total)				
		Overestimation	Correct	Underestimation	Total	*p*-value**
BMI	Underweight	28 (8.9)	5 (1.6)	–	33 (10.5)	< 0.01
	Normal weight	61 (19.2)	80 (25.2)	8 (2.5)	149 (46.9)	
	Overweight	1 (0.3)	64 (20.2)	22 (6.9)	87 (27.4)	
	Obese	–	5 (1.6)	43 (13.6)	48 (15.2)	
	Total	90 (28.4)	154 (48.6)	73 (23.0)	317 (100)^a^	
Age groups	18–25	31 (9.8)	34 (10.7)	7 (2.2)	72 (22.7)	< 0.01
	26–45	32 (10.1)	73 (23.0)	27 (8.5)	132 (41.6)	
	46+	27 (8.5)	47 (14.8)	39 (12.3)	113 (35.6)	
	Total	90 (28.4)	154 (48.6)	73 (23.0)	317 (100) ^a^	

^a^Missing values (*n* = 38), **p-values tested with chi-square

Approximately 76% of the participants had the same self-perceived body size and desired body size, showing no body discrepancy; 5% had a positive discrepancy, while 18% had a negative discrepancy (Table 3). Across the three classifications of discrepancy–negative, none (no discrepancy) and positive–participants with normal weight (*n* = 147; 47%) were the majority; contributing 12, 32 and 3%, respectively. No significant differences were found for age-distribution between negative and positive (p 0.79) or none and positive (*p* = 0.65), the difference between none and negative discrepancy was significant (*p* = 0.007). Further analysis of the participants’ desired body size indicated that both obese women and obese men had no desire to gain weight. Participants who were classified as having normal weight had the highest negative discrepancy (55%) in both genders. Obese and overweight women showed the highest positive discrepancy (33%), indicative of a desire to lose weight, compared to overweight and obese men (9%). No significant differences were found between different age groups (*p* ≥ 0.05).

**Table 3:** Distribution of participants’ body discrepancy for desired body size

		Body discrepancy n (% in total)			
		Negative	None	Positive	Total
Self-perceived body size	Underweight	12 (3.8)	20 (6.3)	1 (0.3)	33 (10.5)
	Normal weight	39 (12.4)	100 (31.7)	8 (2.5)	147 (46.7)
	Overweight	5 (1.6)	79 (25.1)	3 (1.0)	87 (27.6)
	Obese	2 (0.6)	41 (13.0)	5 (1.6)	48 (15.2)
	Total	58 (18.4)	240 (76.2)	17 (5.4)	315 (100)^a^

^a^Missing values (*n* = 40)

**Fig. 3:**
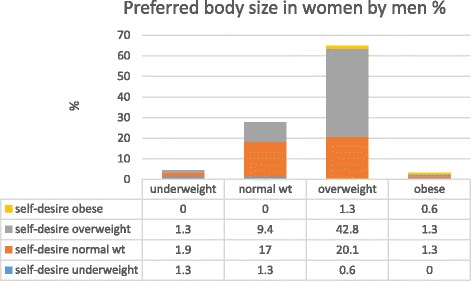
Men’s preferred body size in women according to their self-desired body size

**Fig. 4:**
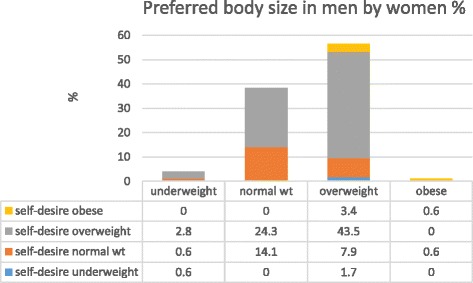
Women’s preferred body size in men according to their self-desired body size

Overall, 65% of men and 57% of women preferred an overweight body size in the opposite gender. However, only 4% of men and 1% of women indicated a preference for an “obese” body size in opposite gender. Figure 3 shows the preferred women’s body size according to men’s self-desired body size. Approximately 43% of the men who desired to be overweight preferred overweight women. No significant differences were found between different age groups (p ≥ 0.05). Figure 4 shows the preferred men’s body size according to women’s self-desired body size. Approximately 44% of the women who preferred overweight men desired to be overweight themselves. Women in this category showed no preference for obese men.

## Discussion

In this study, we found an overall preference for an overweight body size both at the individual level and in the opposite gender, and a significant difference in body size perception associated with age.

The Saharawi refugee population has been settled in the Algerian desert for decades; the refuges are dependent on food aid. Food scarcity is known to contribute to larger body ideals [25–27], and in African and Arabic countries, an increasing trend towards overweight and obesity has been reported [28]. Previously, high rates of overweight and obesity have been documented among the Saharawi refugees [29].

In the present study, the overall prevalence of overweight and obesity was 28% and 15% respectively, with more overweight women than men. While nutrition transition is suggested as one of the main contributors to the change in overweight and obesity, the picture is complicated [30]. Nutrition transition is a well-documented global phenomenon characterized by the adoption of modern diets that are high in fat, sugar, and salt, and the abandonment of traditional diets high in fiber, grains, fruits, and vegetables [30]. Refugees are particularly prone to nutritional transitional changes due to forced migration and reliance on food assistance. Consequently, they usually have a low-diversity diet comprised of starchy foods, refined grains, sugars, and low quantities of fresh or dried fruits and vegetables [31]. In the present population, nutritional transition appears to have given rise to the emergence of a “new” food category primarily based on the Western diet and fast foods [12]. This new food category is more popular among the younger population who may prefer it to food aid and the traditional Sahrawian diet [12].

### Perceived body size

Almost half the participants in the present study accurately perceived their body size. This finding is in accordance with a study on rural dwellers from Nigeria who between the ages of 18 and 71 years [32]. Participants in the youngest age group (18–25 years) were the least likely to underestimate their body size and almost as likely to overestimate their body size in comparison to 26–45 years age group. This trend could be attributed to sociocultural expectations of this age group (18–25 years). During this period, adult Sahrawians usually prepare for and get married. An overestimation of body size was also found in a previous study conducted on Saharawi women living in Morocco, who share similar cultural norms as the sample in the present study, in which the majority of women desired to gain weight, particularly before marriage [33]. The desire to gain weight could also be extended to the men within that age group, since being overweight is associated with health and wellbeing [34]. In addition, the belief that during post-puberty, Saharawi women should have heavier bodies in order to dress in the traditional clothing could be a fueling factor in the overestimation of body size perception among the women [10]. The study revealed that older participants (46 years and older) were the most likely age group to underestimate their body size in comparison to the other age groups. This is consistent with other findings, which show that older adults (50 + years) report having a smaller body size than their actual BMI categorization [35–37].

In addition, although not investigated in this study, the practice of fattening rituals aimed at inducing weight gain among Moroccan Saharawi women could be another possible factor for the acceptance of overweight among the overweight women [10]. In this study, a majority of the participants (77%) who overestimated their body size were men. This finding is in agreement with studies which have shown that men have an incorrect body-size perception, gravitating towards overestimating their body size [38, 39]. Other studies have found the opposite [39, 40] in European culture, where a slim body ideal exists among the men, which is contrary to the body ideal observed among male Saharawi refugees. In the present study, one-quarter of the overweight and obese underestimated their body size. This may suggest that they did not acknowledge their extra weight and the potential risks that it poses. This result is synonymous with findings from a study in Nairobi on adults in which more than half of obese and overweight respondents underestimated their body size [41].

### Body discrepancy

In the study, participants had a higher desire to gain weight in comparison to the desire to lose weight, which could indicate how satisfied they are with their perceived body size. This acceptance of body size is further reflected by the large proportion of participants who showed no body discrepancy. This finding contradicts past research which has demonstrated that, while using figure drawings, women were found to desire an ideal figure that was smaller than their current [42]. On the other hand, in this study, participants that were classified as having normal weight were found to have the highest negative discrepancy, indicating a desire to gain weight across both genders. This result is consistent with findings from a study conducted among Moroccan Saharawi women and other studies in African societies which indicate positive cultural views associated with larger body sizes [33, 41, 43]. In this study, overweight and obese women had a higher positive discrepancy, signaling a higher desire to lose weight in comparison to their male counterparts. This finding contradicted findings from a study examining the sociocultural influences on attitudes towards obesity among Moroccan Saharawi women, in which obese and overweight women had no desire to lose weight [33].

By age, the present study revealed that participants between 18 and 25 years old had the least desire to be overweight or obese in comparison to those who were older. This finding showed the possible effects of acculturation with Western culture (Westernization). Young Sahrawi refugees are often offered two-month summer programs in Spain with host families [12]. While in Spain, albeit for a short time, they are exposed to the Western body ideal, which favors a slim body size [11]. A study on identity among young Saharawi refugees reflected this adoption of preference for the Western, slim, body ideal among young female refugees who spent holidays abroad. This group associated “female beauty” with a slim body and disregarded the plump body size as being part of the old and “bad” side of the traditional life [12]. This, together with a Western body ideal presented in magazines, television shows, advertisements, music television, and popular films, could be a probable cause of change in attitude and redefinition of ideal body size among young Saharawi refugees.

### Preferred body size in the opposite gender

Body size preference is a dynamic interaction between family, community, and economic systems [44]. In this study, both women and men preferred overweight in the opposite gender. This finding is in agreement with previous studies that indicate a preference for an overweight body ideal in many African countries as it is indicative of attributes such as health, fertility, beauty, wealth, and power [25, 34, 37, 38, 41, 43, 45]. The self-perceived body-size by a woman is affected by the male’s desired size for female and vice versa; however, both genders might be exaggerating the opposite-sex preferences, which leads to misjudgments about attractive body sizes. For example in western countries, when women were asked to select men’s ideal body size, they selected a much thinner figure than what men select as ideal, and men selected a more muscular figure than what was desired by women. [46].

### Strengths and limitations

The strength of this study is a large, randomly selected sample including both men and women. A limitation of the study is the use of body figure scales in studies on body size preferences. In this study, we linked BMI to the body figures and made four categories (Fig. 1); as such, the body figures in each category are not differentiated. From body figure IV to V, there is an interval of two units from 23 to 25 kg/m^2^. Individuals with a BMI of 24 kg/m^2^ will therefore have a body size that does not correspond to any of the body figures. This individual may then be wrongly classified as overweight. However, in previous studies where BMI has been linked to the figures, BMI correlates with self-perceived body size [21, 22]. Finally, it is good to use figure scales as a cost-effective method in epidemiological studies [47].

## Conclusion

This study on body-size perceptions and preferences among adult Saharawi refugees revealed inter-generational differences in the way the refugees perceived body size. There was a dominant preference for an overweight body size both at the individual level and in the opposite gender. Whereas women had a higher prevalence of overweight, men overestimated their body size towards being overweight. The youngest refugees had the least desire to gain weight in comparison to the older refugees. In spite of this, there seemed to still exist strong cultural expectations in favor of a bigger body size.

## Abbreviations

BMI: Body mass index
WHO: World Health Organization
